# Editors’ Message – September 2024

**DOI:** 10.1016/j.ajpc.2024.100729

**Published:** 2024-08-30

**Authors:** Nathan D. Wong, Erin D. Michos

**Affiliations:** a Division of Cardiology, School of Medicine, University of California, Irvine, CA, USA; b Division of Cardiology, Johns Hopkins University School of Medicine, Baltimore, MD, USA

We hope you have all enjoyed your summer! In this issue of the *American Journal of Preventive Cardiology (AJPC),* we are pleased to share several updates with you that continue to advance the progress of *the AJPC* as the key forum for the publication of content in preventive cardiology. As we have said before, the continued progress of the *AJPC* would not have been possible without the dedicated hard work of our associate editors, editorial board, and of course our devoted authors who have believed in the AJPC since its beginning more than four years ago.

Most importantly, we are pleased to announce that while over 90 % of journals this past year have seen a decrease in impact factor, the *AJPC* saw an increase in impact factor to 4.3, placing it in nearly the top quintile of all journals in the cardiovascular medicine field (ranking 45/220, or at the 79.8th percentile). Also notable is that the top four journals citing the *AJPC* included *Circulation,* the *Journal of the American College of Cardiology, New England Journal of Medicine,* and *JAMA*.

With a solid impact factor, the *AJPC* continues to attract more and more submissions. Just through July 2024, we have had approximately 175 submissions, more than the 134 for the entire year of 2023. Consequently, this has required greater selectivity in the review process with a higher bar required to achieve acceptance of an article. Further, to ensure efficient processing of journal articles, we continue to increase our editorial board. We are pleased to welcome our first associate editor based in Latin America, Dr. Daniel Piskorz from Rosario, Argentina. In addition, we have recently added new editorial board members, including Dr. Barry Franklin from Royal Oak, Michigan, Dr. Anandita Kulkarni from Plano, Texas, John William McEnvoy from Galway, Ireland, Dr. Priyanka Satish from Austin, Texas, Dr. Leandro Slipczuk from Bronx, New York, and Dr Brittany Weber from Boston, Massachusetts. We look forward to their great work with the *AJPC*.

This issue of the *AJPC* is dominated by original research contributions. Some key features include articles on how epicardial fat modifies the relation of coronary calcium score with mortality, a retrospective analysis of testing practice and clinical management of lipoprotein(a), trends in cigarette smoking and incident cardiovascular disease among Asian and Pacific Islanders, ezetimibe use and mortality after myocardial infarction in a national cohort, and an article on cardiovascular risk and subclinical atherosclerosis in first degree relatives of patients with premature cardiovascular disease. Other interesting contributions include the projected impact of the inflation reduction act's climate provisions on cardiovascular and respiratory outcomes, relationship between epicardial adipose tissue and coronary atherosclerosis, home-based cardiac rehabilitation and mortality and hospital readmission rates, primary care clinician engagement in implementing a machine-learning algorithm for targeted screening of familial hypercholesterolemia, and on sex and gender aspects influencing non-adherence to secondary prevention measures. Commentaries on advances in predicting cardiovascular risk applying the PREVENT equations, bempedoic acid in secondary prevention, as well as short reports on the impact of a specialized cardiometabolic clinic and on disparities in awareness and treatment among women with hypertension from the American Heart Association Research Goes Red Registry are also included.

The *AJPC* looks forward to continued submissions of high-quality research and review articles from the many experts in the field of preventive cardiology. We look forward to continuing to hear from you, our dedicated readership, on how we can strive to continue to make the *AJPC* the place to go to publish work on the latest advances in preventive cardiology!

## Author agreement

The authors have agreed to the submission of this manuscript – Editors’ Message – September 2024 and the materials in this manuscript have not been previously published nor are in consideration for publication elsewhere.

## CRediT authorship contribution statement

**Nathan D. Wong:** Writing – original draft. **Erin D. Michos:** Writing – review & editing.

